# Evaluation of treatment with a combination of mycophenolate mofetil and prednisolone in dogs with meningoencephalomyelitis of unknown etiology: a retrospective study of 86 cases (2009–2017)

**DOI:** 10.1186/s12917-020-02414-3

**Published:** 2020-06-12

**Authors:** Joong-Hyun Song, Do-Hyeon Yu, Hee-Chun Lee, Tae-Sung Hwang, Young Joo Kim, Su-Jin An, Dong-In Jung

**Affiliations:** 1 Institute of Animal Medicine, College of Veterinary Medicine, Gyeongsang National University, Jinju, 52828 South Korea; 2 College of Veterinary Medicine, Western University of Health Sciences, Pomona, California, 91766-1854 USA

**Keywords:** Granulomatous meningoencephalitis (GME), Meningoencephalomyelitis of unknown etiology (MUE), Mycophenolate mofetil (MMF), Necrotizing meningoencephalitis (NME), Prednisolone

## Abstract

**Background:**

Combination therapy with glucocorticoids and adjunctive immunomodulating drugs has been generally accepted as a standard treatment regimen for meningoencephalomyelitis of unknown etiology (MUE). We hypothesized that treatment with MMF as an adjunctive agent along with glucocorticoids would be effective and well-tolerated protocol in dogs with MUE. Eighty-six dogs with MUE between May 2009 and June 2017 were included (59 females and 27 males; mean age of 5.93 years; mean body weight of 3.83 kg). The medical records of dogs with MUE treated with prednisolone and MMF were retrospectively evaluated to determine the therapeutic response, survival time, and treatment-related adverse effects.

**Results:**

A partial or complete response (CR) was recorded for 75 dogs. The overall median survival time from the initiation of treatment was 558 days. Dogs that showed CR with no relapse over the treatment period (from diagnosis to death) had significantly longer median survival times. A significantly higher mortality hazard ratio of 4.546 was recorded in dogs that failed to achieve CR. The interval between the onset of clinical signs and the clinical presentation was not significantly associated with CR, relapse rate, and survival time. Adverse effects included gastrointestinal upsets in 26 dogs (30.23%), sporadic infections in 17 dogs (19.77%), and pancreatitis in seven dogs (8.14%).

**Conclusions:**

The results suggest that adjunctive MMF treatment for MUE is safe and comparable to other immunosuppressive protocols. The treatment should focus on the achievement of CR and preventing relapse for successful management.

## Background

Non-infectious inflammatory disorders of the central nervous system (CNS) can be divided into several subtypes based on the characteristics of the affected animal and the specific histopathological features. Among these disorders, granulomatous meningoencephalomyelitis (GME), necrotizing meningoencephalitis (NME), and necrotizing leukoencephalitis (NLE) have been frequently reported [1]. The umbrella term meningoencephalomyelitis of unknown etiology (MUE) has been introduced to describe dogs with non-infectious CNS inflammatory disorders without a histopathological diagnosis [2].

MUE has long been assumed to have an autoimmune and genetic pathogenesis, and it seems to involve aberrant immune responses directed against CNS constituents [3]. Many glucocorticoid-based protocols have been investigated for the treatment of MUE. Glucocorticoid monotherapy may adequately control the clinical signs initially, but the long-term response is variable, and immunosuppressive doses of glucocorticoids may cause adverse systemic effects in some patients [4, 5]. For these reasons, combination therapy with glucocorticoids and one or more immunomodulating drugs, or monotherapy with immunomodulating drugs has been generally accepted as the standard treatment regimen [1].

Mycophenolate mofetil (MMF) is the pro-drug of mycophenolic acid, an inhibitor of the enzyme inosine monophosphate dehydrogenase (IMPDH) [6]. It has multiple immunosuppressive effects, which are exerted mostly through activated lymphocytes. Lymphocytes are completely dependent on de novo purine biosynthesis, and the de novo synthesis of guanosine monophosphate for purines requires IMPDH. Therefore, inhibition of IMPDH by MMF leads to purine depletion specifically within lymphocytes and ultimately results in inhibited DNA production and cytostatic activity on both activated B- and T lymphocytes [7].

MMF has been used in veterinary medicine as an immunosuppressant to prevent allograft rejection and for various immune-mediated diseases [8–13]. It has also recently been considered a comparable alternative to other published immunosuppressive agents in the treatment of MUE [14–17]. The most commonly reported adverse effects of MMF are gastrointestinal upsets; however, these adverse effects were infrequent, transient, and non-life threatening in most cases [10, 12, 14, 17]. The purported advantages of using MMF over other adjunct immunosuppressive drugs include its rapid onset of action, availability in oral and parenteral formulations, and lack of myelotoxicity or hepatotoxicity [12, 18].

The purpose of this retrospective study was to describe the use of MMF as an adjunctive immunosuppressive agent administered with glucocorticoids in client-owned dogs with MUE. We hypothesized that treatment using MMF as an adjunctive agent with glucocorticoids would be effective and well-tolerated protocol in dogs with MUE. Furthermore, we aimed to report any adverse effects of long-term MMF therapy and risk factors associated with the clinical outcomes in dogs with MUE. In addition, we also tried to improve our understanding of MUE treatment through various analyzes with more strict medication control and large-scale filed than in previous studies.

## Results

One-hundred and eight dogs were included on the basis of the initial collection criteria. After assessments, 22 dogs were excluded for the following reasons: discontinuation of the MMF treatment (*n* = 8); receiving additional immunosuppressants (*n* = 4); lack of cerebrospinal fluid (CSF) evaluation data (n = 4); and lack of follow-up data (*n* = 6). Finally, 86 dogs were included in the study.

### Signalments, clinical signs and treatment variations

Affected breeds included the Maltese (*n* = 36), Shih Tzu (*n* = 9), Yorkshire Terrier (n = 9), Chihuahua (*n* = 6), Pomeranian (n = 6), mixed-breed (*n* = 5), Toy Poodle (*n* = 3), Miniature Pinscher (n = 3), Pekinese (n = 3), and Miniature Dachshund (*n* = 2), and one each of Papillon, Shetland Sheepdog, Miniature Schnauzer, and French Bulldog. There were 59 females (13 spayed and 46 sexually intact) and 27 males (16 castrated and 11 sexually intact). Age at initial diagnosis ranged from 7 months to 15 years (median, 6 years; mean, 5.93 years). Body weight ranged from 1.45 to 11.7 kg (median, 3.125 kg; mean, 3.83 kg). The median interval from the onset of neurologic dysfunction to initial presentation was 14 days (range, 0–1080 days). Fifty-seven dogs (66.28%) belonged to the acute group and 29 dogs (33.72%) belonged to the chronic group.

At presentation, all dogs had clinical signs of neurologic dysfunction. Signs included seizure (*n* = 38), ataxia (*n* = 22), circling (*n* = 11), vestibular dysfunction (n = 11), hemiparesis (*n* = 10), tetraparesis (*n* = 9), paraparesis (*n* = 8), changes in mental status (e.g., dullness, restless or aggression) or altered behavior (*n* = 7), blindness (n = 7), head turn (*n* = 6), kyphosis (*n* = 2), painful reaction (n = 2), and cerebellar ataxia (*n* = 1).

Anti-epileptic drugs were initially administered to all dogs that presented with serial seizure episodes (23/86, 26.74%), 13 of which were prescribed phenobarbital (2 to 2.5 mg/kg PO every 12 h), 6 of which were prescribed potassium bromide (40 mg/kg PO every 24 h) and 4 of which were prescribed zonisamide (5 mg/kg PO every 12 h). Cefixime (5 mg/kg PO every 12 h) and metronidazole (10 mg/kg PO every 12 h) were also initially administered to 3 dogs (3/86, 3.45%) suspected infectious meniningoencephalomyelitis, and was immediately ceased when negative PCR results were identified.

### Diagnostic imaging findings

Abnormal findings on diagnostic imaging (computed tomography [CT], *n* = 41; magnetic resonance imaging [MRI], *n* = 45) were detected in all dogs. Forty-five dogs (52.33%) showed a focal lesion and 41 dogs (47.67%) showed multifocal lesions. The affected neuroanatomic sites included the prosencephalon (*n* = 72), brainstem (*n* = 23), spinal cord (n = 2), and cerebellum (*n* = 1). Eleven (12.8%) of the dogs had both prosencephalon and brainstem lesions and only one (1.16%) dog had both prosencephalon and cerebellum lesions.

### CSF analysis findings

The CSF was analyzed in all 86 dogs. Pleocytosis was detected in 79 of 86 dogs (91.86%) at the initial diagnosis. The median total nucleated cell count (TNCC) was 12 cells/μl (mean, 31.09 cells/μl; range, 1–372 cells/μl). All abnormal samples predominantly showed mononuclear cell (with predominance of either lymphocytes or macrophages) pleocytosis. Total protein (TP) concentration in the CSF was elevated in 52 of 86 dogs (60.47%), and the median TP concentration was 30 mg/dl (mean, 32.17 mg/dl; range, 0–300 mg/dl). Infectious disease testing using a real-time PCR assay of the CSF was performed in 15 of 86 dogs. The results for the PCR tests were negative in all 15 samples. All CSF samples were also examined microscopically and were all negative for infectious agents.

### Response to therapy and survival time

Complete response (CR) after treatment initiation was recorded in 57 dogs (66.28%), whereas 18 dogs (20.93%) showed partial response (PR) and 11 dogs (12.79%) were unresponsive (no response [NR]) to therapy. CR within a month of treatment initiation was recorded in 49 dogs (56.98%). There was no statistically significant difference in CR rates between the acute group and the chronic group (*P* = 0.596). In a univariate multiple data analysis used to identify the risk factors for CR probability (Table 1), a significantly higher odds ratio was recorded in dogs that showed neurological dysfunctions other than only seizure history before presentation, relative to those that had only seizure history (odds ratio of 3.701; *P* = 0.013; 95% confidence interval, 1.311–10.488). Of the 75 responders (both CR and PR), 34 dogs (45.33%) showed a relapse after achieving a treatment response. The relapsed dogs were managed successfully with an increased dose of prednisolone or MMF, and some patients needed additional treatment with anticonvulsants (16/34, 47.06%). Of the 34 relapsed dogs, only five dogs (14.7%) failed to achieve re-remission of the relapsed clinical signs. There were no significant (*P* = 0.302) differences in relapse rates between the acute group and the chronic group.

**Table 1:** Results of univariate multiple logistic analysis of potential risk factors for failed CR among dogs treated with a combined MMF-prednisolone therapy

Factor	CR, % (n)	Failed CR, % (n)	*P*
Sex			
Male	66.67 (18)	33.33 (9)	0.789
Female	66.1 (39)	33.9 (20)	
Age			
< = 7 years	61.67 (37)	38.33 (23)	0.083
> 7 years	76.92 (20)	23.08 (6)	
Lesion distribution			
Focal	75.56 (34)	24.44 (11)	0.087
Multifocal	56.1 (23)	43.9 (18)	
Brainstem involvement			
Negative	73.33 (44)	26.67 (16)	0.179
Positive	50 (13)	50 (13)	
Seizure			
Negative	53.49 (23)	46.51 (20)	0.393
Positive	79.07 (34)	20.93 (9)	
Seizure as the only symptom			
Negative	55.77 (29)	44.23 (23)	0.013^a^
Positive	82.35 (28)	17.65 (6)	
Body weight	–	–	0.758
TNCC	–	–	0.162
Symptom duration before treatment	–	–	0.568

^a^odds ratio, 3.701; 95% confidence interval, 1.311–10.488

*CR* complete response, *TNCC* total nucleated cell count

**Fig. 1:**
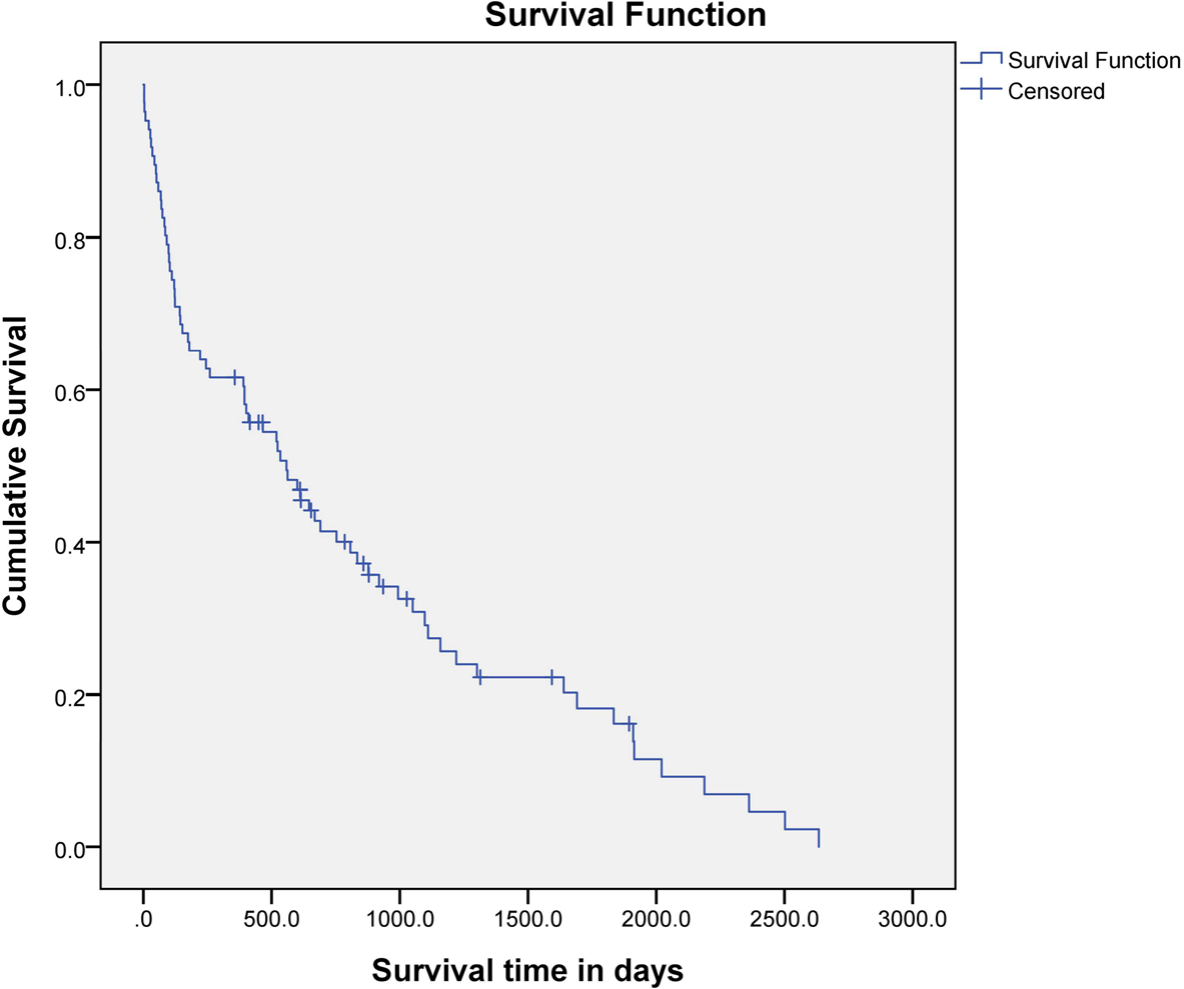
Kaplan-Meier survival curve for 71 dogs treated with MMF and prednisolone for MUE. The MST was 558 days (range, 3–2634 days)

**Fig. 2:**
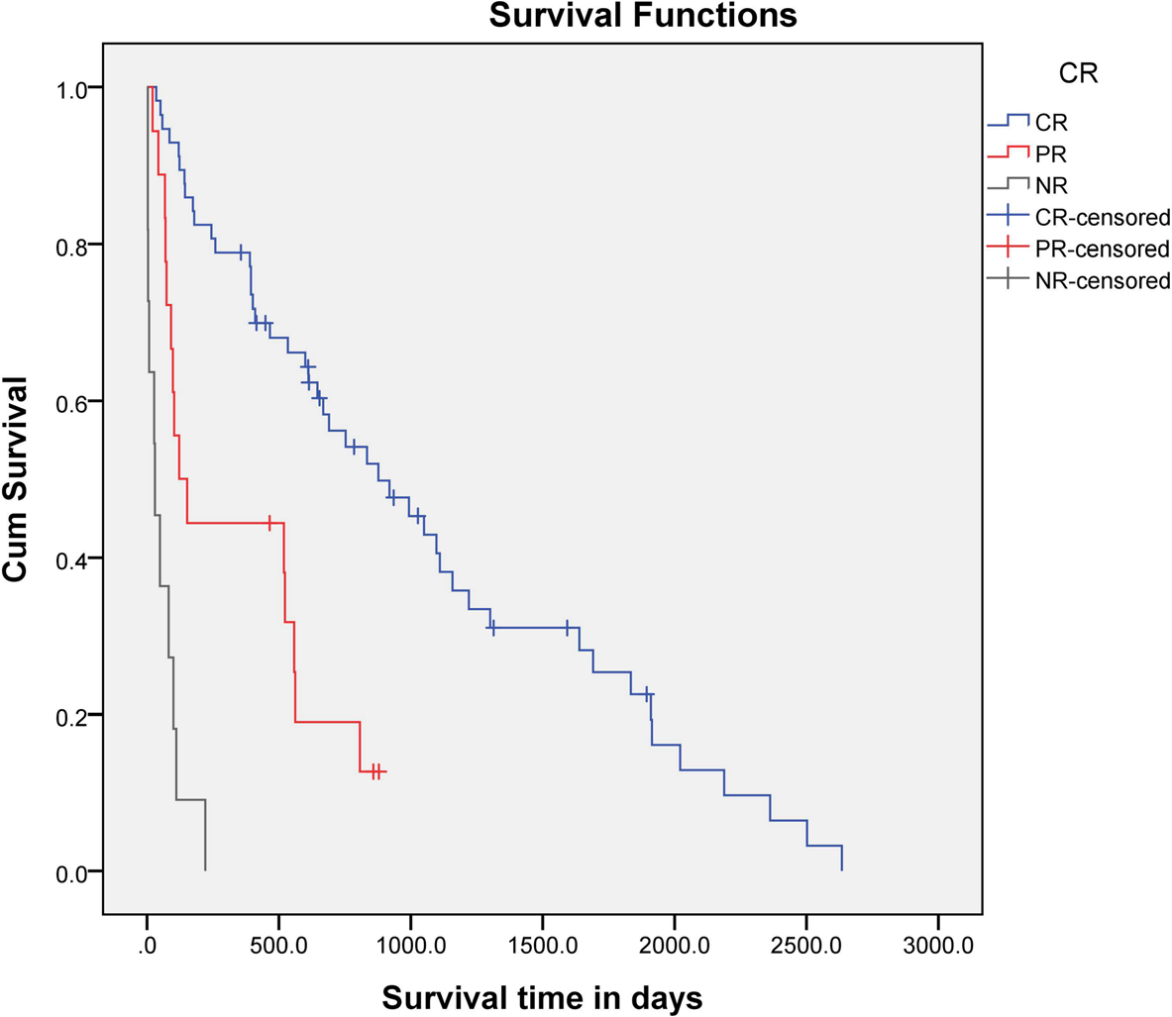
Kaplan-Meier survival curve for 71 dogs treated with MMF and prednisolone for MUE in the CR (*n* = 45), PR (*n* = 15), and NR (*n* = 11) groups. Survival times differed significantly between the groups

**Fig. 3:**
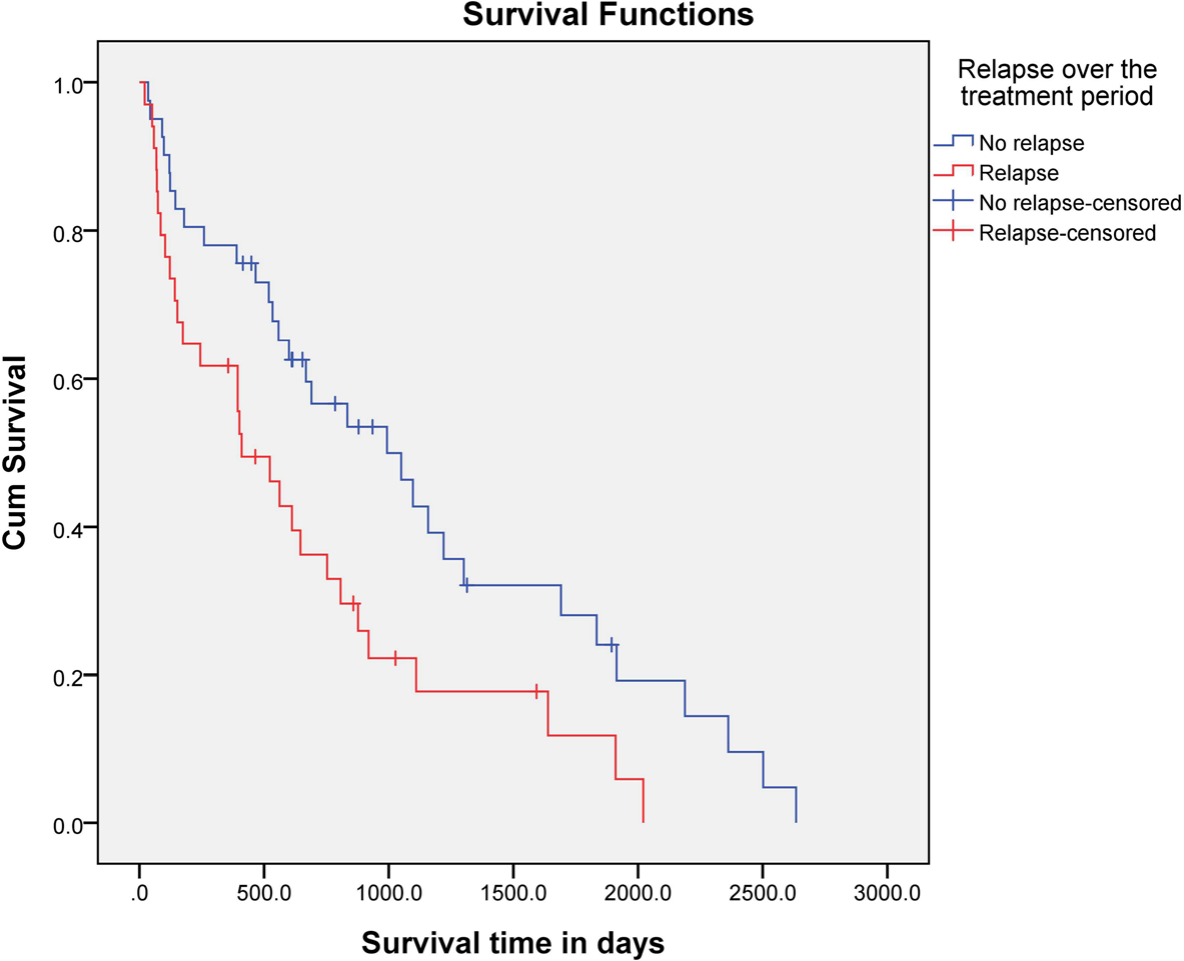
Kaplan-Meier survival curves for 60 dogs treated with MMF and prednisolone for MUE. Dogs that did not show relapse within the study period (*n* = 31) were compared with those that did show relapse (*n* = 29). There was a significant difference between both groups (*P* = 0.011)

The median survival time (MST) for all dogs was 558 days (range, 3–2634 days) (Fig. 1). Fifteen dogs (17.44%) were censored from the survival analysis; 13 dogs were still alive at the end of the study, one dog died from concurrent heart disease, and the other dog died from concurrent pancreatitis. The MST for dogs in the acute group was 401 days (range, 3–2502 days) and that for dogs in the chronic group was 807 days (range, 3–2634 days). There was no statistical difference (*P* = 0.125) in survival time between the acute and chronic groups. There were significant (CRs compared with PRs, *P* <  0.001; CRs compared with NRs, P <  0.001; PRs compared with NRs, *P* < 0.001) differences in the MST for dogs that showed a CR to treatment during the study period (877 days; range, 35–2634 days), compared with the MST for dogs that showed a PR (122 days; range, 21–879 days) or NR (30 days; range, 3–221 days) (Fig. 2). Furthermore, the MST of the dogs that showed CR within a month (877 days; range, 35–2634 days) was significantly (*P* = 0.004) longer than that of the dogs that did not (111 days; range, 3–2502 days). The MST was also significantly (*P* = 0.011) longer for dogs that did not show relapse (993 days; 35–2634 days) than for dogs that did show relapse (410 days; range, 21–2021 days) (Fig. 3).

**Fig. 4:**
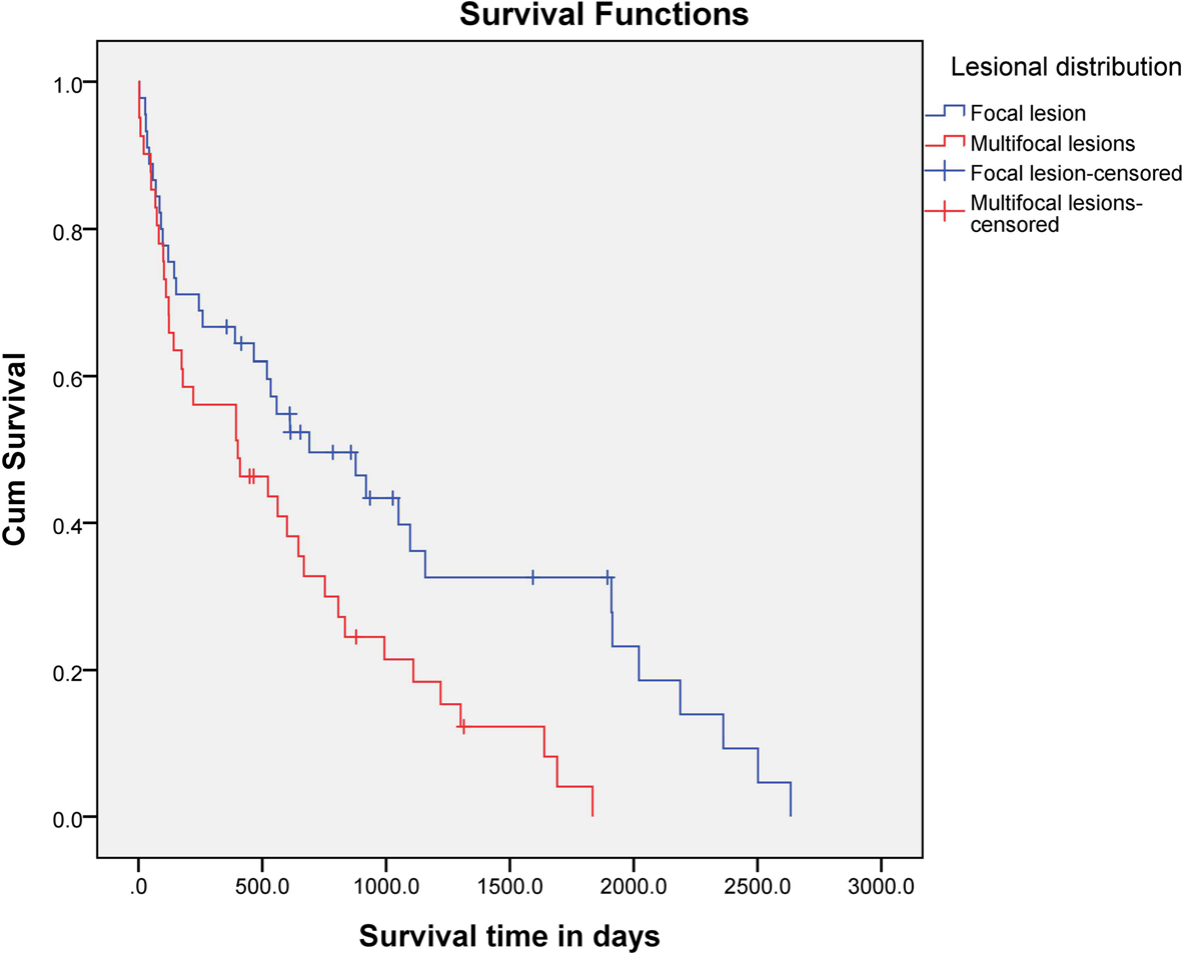
Kaplan-Meier survival curves for 71 dogs treated with MMF and prednisolone for MUE. Dogs that showed a focal lesion (*n* = 34) were compared with those showing multifocal lesions (*n* = 37). There was a significant difference between both groups (*P* = 0.01)

**Fig. 5:**
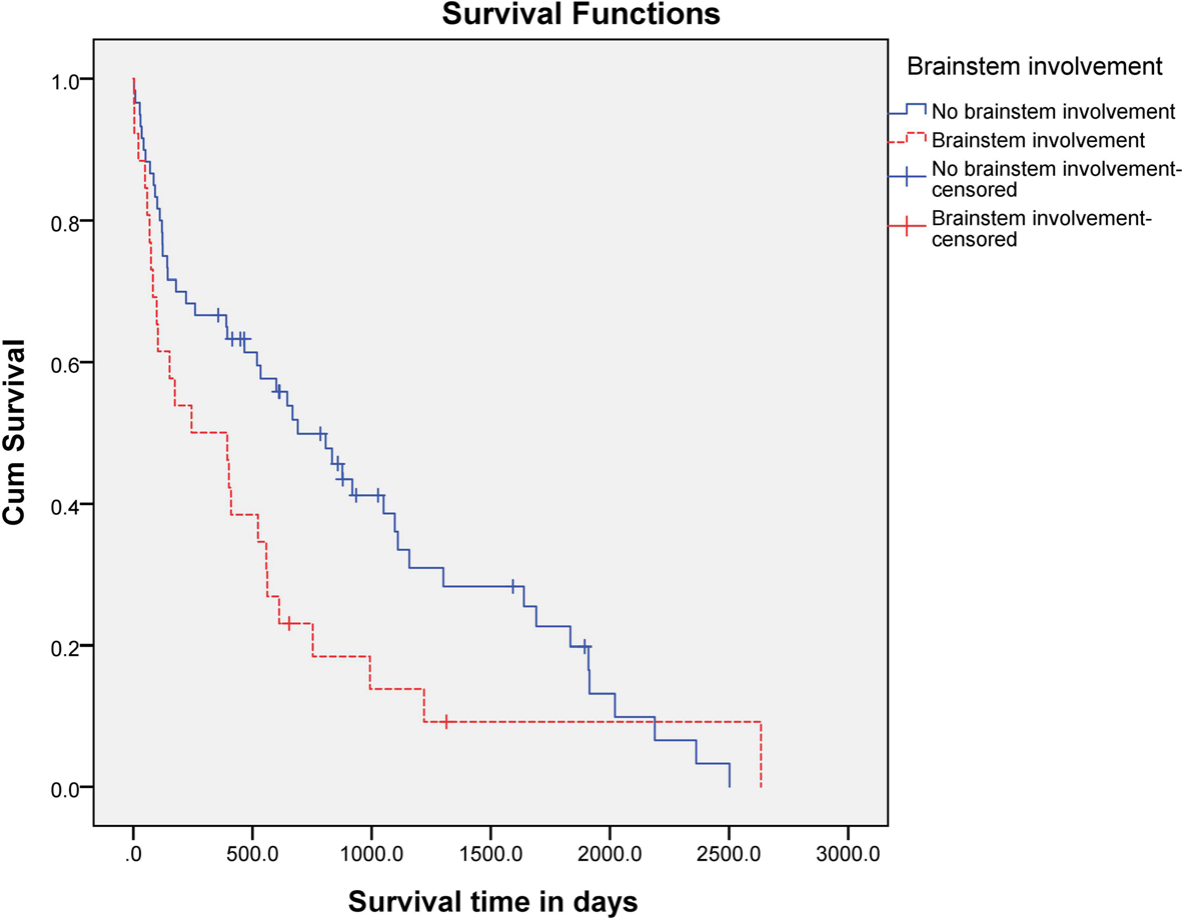
Kaplan-Meier survival curves for 71 dogs treated with MMF and prednisolone for MUE. Dogs that showed brainstem involvement (*n* = 24) were compared with dogs that did not show brainstem involvement (*n* = 47). There was no statistical difference in MST between both groups (*P* = 0.057)

The MST was significantly (*P* = 0.01) longer for dogs that had a focal lesion (690 days; range, 4–2634 days) than for dogs that had multifocal lesions (401 days; range, 3–1834 days) (Fig. 4). The MST for dogs with brainstem involvement was 244 days (range, 3–2634 days) and that for dogs that did not have brainstem involvement was 609 days (range, 3–2502 days). There was no statistical difference in MST between the two groups (*P* = 0.057) (Fig. 5).

Univariate multiple Cox regression analysis with 10 variables showed that the CR and TNCC at diagnosis were significant factors in predicting mortality (Table 2). The dogs that failed to achieve CR (both NR and PR) over the study period had a 4.546-fold higher relative hazard ratio of mortality than those who achieved CR (*P* < 0.001; 95% confidence interval, 2.596–7.960). The TNCC at the diagnosis increased the relative hazard ratio for mortality by 1.004-fold with an increase in cell count (*P* = 0.032; 95% confidence interval, 1.000–1.008). Age, sex, body weight, the presence of seizure history, the duration of clinical signs before treatment, lesional distribution, brainstem involvement, and TP had no impact on mortality.

**Table 2:** Univariate multiple Cox regression analysis showed the CR and TNCC at diagnosis were significant factors in predicting mortality

Variables	Relative hazard ratio	*P*	95.0% confidence interval	
			Lower	Upper
CR	4.564	< 0.001	2.596	7.960
TNCC	1.004	0.032	1.000	1.008

### Treatment-related adverse effects

Treatment-related adverse effects were reported in 39 of 86 (45.35%) dogs. Of these 39 cases, 14 dogs had two or more concurrent adverse effects. The most common adverse effect was gastrointestinal problems (*n* = 26 dogs, 26/86, 30.23%); we censored any patients with gastrointestinal manifestations associated with extra-gastrointestinal diseases (e.g., infectious diseases) and pancreatitis; these included diarrhea (*n* = 16, 3 of 16 dogs had hemorrhagic diarrhea), vomiting (*n* = 11, 1 of 11 dogs had hemorrhagic vomiting), anorexia (*n* = 3), and constipation (n = 1). Most of these dogs showed gastrointestinal signs within a month after the initial treatment. Some dogs required supportive therapy, but most symptoms were resolved with a reduced dose of MMF. The dose of MMF was reduced by 50% from that of the pervious dose and slowly increased up to the maximum tolerated dose over one month. The others required no treatment and resolved themselves within a few days. The second most common adverse effect was sporadic infections (*n* = 17 dogs, 17/86, 19.77%); these included pyometra (*n* = 4), otitis externa (*n* = 3), bacterial dermatitis (*n* = 2), fungal dermatitis (n = 2), demodicosis (n = 2), bacterial rhinitis (n = 2), urinary tract infection (n = 2), and one each of bronchopneumonia, vaginitis, and mastitis. Infectious diseases were treated with an appropriate therapy for each infectious agent and pyometra was resolved with surgical intervention. Pancreatitis was reported in seven dogs (7/86, 8.14%), and one of these dogs had concurrent pancreatic abscess. Pancreatitis was resolved in most patients with supportive therapy, and the dog that had a pancreatic abscess underwent surgical treatment. One of these dogs was euthanized by the owner due to the recurrent chronic pancreatitis. Adverse effects attributable to prednisolone therapy were not recorded in this study and improved during tapering.

Hematologic abnormalities were identified in 49 dogs (49/86, 56.98%). Abnormal results of CBC were found in 26 of 86 (30.23%). Abnormal results of biochemistry were found in 34 of 86 (39.53%). Of these, 16 dogs had two or more concurrent hematologic abnormalities. These included elevated liver enzyme activity levels (*n* = 27), leukocytosis (*n* = 17), thrombocytosis (*n* = 10), anemia (*n* = 8), hypertriglyceridemia (*n* = 6), elevated lipase activity (*n* = 3), hypercholesterolemia (n = 3), hyperglycemia (n = 3), and hypocalcemia (n = 1). Most of the adverse effects might have been related to the concurrent administration of glucocorticoids. The issue was mostly resolved with prednisolone tapering, and none of the patients had clinical signs of these hematologic abnormalities. Anemia was mild and transient in all eight dogs. Seven of the dogs had concurrent gastrointestinal bleeding or one of the inflammatory diseases mentioned above, such as hemorrhagic diarrhea, pancreatitis, infectious skin disease, and pyometra. The anemia of the other dog was non-specific and resolved on the next follow-up visit. The patient with hypocalcemia had a medical history of concurrent acute pancreatitis, and hypocalcemia resolved during the recovery of pancreatitis.

## Discussion

The results of this study suggest that the administration of MMF in combination with prednisolone could serve as an immunosuppressive protocol for the initial therapy of MUE that was comparable to the other reported protocols. Most dogs responded to the MMF and prednisolone protocol, with 66.28% of dogs showing a CR. The overall MST after initiating the treatment was 558 days, and 13 dogs (15.12%) that were still alive at the end of the study had a relatively long follow-up time (median, 914 days from treatment; range, 449–2595 days).

The survival times for dogs in this study were similar to those in the previous studies of dogs treated with several immunosuppressive protocols for MUE. In two retrospective studies investigating the use of MMF in combination with corticosteroids, the MSTs were reported to be 250 days (*n* = 20; range, 6–1679 days) [5] and 731 days (*n* = 25; range, 582–1672 days) [17]. In studies of treatment protocols using corticosteroids in combination with other immunosuppressive drugs, the MSTs ranged from 26 to 1834 days (median, 531 days). The MSTs of dogs treated with corticosteroids in conjunction with cytosine arabinoside were 531, 26, and 735 days [7, 19, 20], while the MSTs of those treated with lomustine were 457 (GME) and 329 (necrotizing encephalomyelitis) days [21], and those of dogs treated with cyclosporine were 930 and 620 days [22, 23]. The dogs treated with azathioprine in combination with prednisone had an MST of 1834 days [24], and the dogs treated with procarbazine and prednisone had an MST of 425 days [25]. Some of the studies that showed a large gap in the MSTs against the others showed an obvious difference in study design. In one study of cytosine arabinoside [19], 33% of the enrolled cases died within zero to three days of diagnosis. This suggests that the cases enrolled in this study were unlikely to have a long survival time regardless of the treatment protocol. In the study with azathioprine that showed an MST of 1834 days [24], azathioprine therapy was delayed until after ruling out the possibility of infectious CNS disease. Some dogs that did not survive the intermediate period before obtaining the results for the infectious disease titer were excluded from this study. In one study of MMF therapy [17], additional immunosuppressive drugs were added during the study period and the MST was calculated before the initiation of MMF treatment. Thus, it was concluded that the immunosuppressive protocols reported so far have similar prognoses and the establishment of a gold standard protocol is difficult as of now. Therefore, an individual-based protocol considering the patient’s state, drug availability, affordability, and adverse effects might be the best treatment option for dogs with MUE.

The MST was significantly longer for the dogs that showed a CR compared with the dogs that showed a PR and NR, and most of the patients who showed a CR achieved their responses within a month. In addition, a significantly higher mortality hazard ratio of 4.546 was recorded in dogs that failed to achieve a CR, relative to those that showed a CR over the study period. Thus, these results combined with the results of two other previous studies of MUE [14, 24] suggest that the achievement of CR and improvement within a month were crucial for predicting the prognosis, and clinicians should do their best to achieve CR. Moreover, the presence of neurological dysfunctions other than only seizure history can be used as a good predictor of the failure to achieve a CR in the clinical setting, according to the logistic analysis. The dogs in this study were divided into two groups according to the acuteness of the disease course, based on the current literature [15]. The acuteness had no significant effect on the MST and the CR rates in this study, contrary to the authors’ expectation and a previous study that performed a similar comparison [14]. Although it was not statistically significant, the relatively short MST of the acute group compared to the chronic group indicated that the acute group might have a more severe disease course and may need a more aggressive treatment protocol for a favorable prognosis. However, obvious limitations are evident in this comparison due to the fact that these subgroups were characterized in only a time-dependent manner; further studies based on the severity of the neurological dysfunctions before treatment will be needed.

One previous study of MUE treated with a combined prednisone-azathioprine therapy showed that the MST (472 days) was significantly (*P* < 0.001) shorter for dogs that did relapse than for dogs that did not relapse (1961 days) [24], in agreement with the results of this study. It was thus verified that dogs that did not relapse during treatment had a more favorable long-term outcome. Furthermore, most of the relapsed dogs in both studies showed a favorable response as the dose of corticosteroids was re-increased. This may mean that corticosteroid therapy is one factor contributing to the maintenance of clinical responses and prevention of relapse. Therefore, it seems that tapering of the corticosteroids should be done more cautiously and slowly, but proper drug dosing adjustments in patients with MUE that had shown remission remain elusive.

Signalment data were recorded to evaluate the association of signalment with MUE. Female dogs were over-represented (68.6%), which is comparable to former large-scale, multi-purpose retrospective MUE studies, and the predominance of female patients is considered to be similar to the findings for other immune-mediated diseases [1, 3, 26–28]. Most cases in this study were small breeds and only two of the 86 dogs were medium-sized breed dogs, which was consistent with the results of previous surveys [3, 29]. The Maltese dog (41.86%) was the most commonly affected breed in this study. Additional commonly affected breeds were the Shih Tzu (10.47%) and Yorkshire Terrier (10.47%). Given the fact that the Maltese is one of the most numerous dogs in South Korea [30], MUE might be a prevalent neurological disease in South Korea.

In addition to the MMF-prednisolone therapy, 23 dogs in our study were received anti-epileptic medication as an initial therapy. Most of them received anti-epileptic medication throughout the entire study period. While, the initial administration of anti-epileptic medication with the MMF-prednisolone therapy did not significantly affect patient survival (*P* > 0.05). This result suggests that the anti-epileptic medication does not seem to affect the treatment of MUE itself. Because anti-epileptic drugs frequently used with immunosuppressive treatment in dogs with MUE, however, further well-controlled studies will be needed to clarify the effects of anti-epileptic medication on MUE.

Gastrointestinal problems are well-known MMF-related adverse effects, which are comparatively benign and reversible. Gastrointestinal problems were found in 30.23% of the dogs in this study, and most of them occurred within the first month of treatment. The MMF-prednisolone protocol used in this study was relatively safe with proper management, and there was no need to stop this treatment due to the gastrointestinal upsets. The previous two studies of MUE treated with a combined MMF-prednisone protocol also documented treatment-related gastrointestinal problems. The results from one study showed a 20% rate of gastrointestinal side effects, which was similar to that in our study [14]. Another study showed a relatively low gastrointestinal side effect rate of 8% with similar patient survival as compared with the results of our study [17]. Although there was a difference in the enrolled population, the biggest difference between the studies was the initial dose of MMF. The initial dose of MMF in our study was 20 mg/kg every 12 h based on the previous literature [15], but the other study chose an initial dose of 10 mg/kg every 12 h. This suggests that MMF has dose-dependent gastrointestinal adverse effects, and the dose of 20 mg/kg every 12 h might be high in some patients. Individualized dose-titration or preliminary gastrointestinal protection thus might be required for dogs that need high doses of MMF, especially in the early phase of treatment. In addition, the starting dosage of MMF does not seem to have an overt effect on survival time. This impression is supported by data from the human study of MMF that reported that the adverse gastrointestinal effects of MMF are mainly related to its peak serum concentration [31].

Sporadic infections were not an uncommon adverse effect of long-term MMF therapy in this study. Opportunistic infections are important adverse effects related to the long-term immunosuppressive therapy used in immune-mediated diseases. They are commonly managed by appropriate therapy, but when they are not treated in a timely manner, they can cause a fatal illness. Given the relatively high incidence of sporadic infections in this study, regular check-up examinations and the maintenance of a high level of sanitation should be pursued for the long-term management of dogs treated with MMF.

Corticosteroids have long been considered to be a risk factor for canine pancreatitis [32, 33]. However, this belief has not been proven and is now largely dismissed by recent works [34]. While pancreatitis was recorded in 8% of the dogs in this study, no previous reports have documented the incidence of pancreatitis after MMF treatment in veterinary medicine. Two previous studies of human patients have suggested the possibility of MMF-induced pancreatitis [35, 36]. The mechanism underlying MMF-induced pancreatitis remains unknown, but an allergic response to pancreas was proposed, like azathioprine, which has a similar mechanism of immunosuppressive action to that induced by MMF [37, 38]. Thus, this long-term study has suggested pancreatitis as a new adverse event of MMF treatment in canine patients, and further investigation will be needed to confirm the association of pancreatitis with the combined MMF-corticosteroids therapy.

There are several limitations in this study. First, histopathological confirmation of the diagnosis was not performed in most cases. Although the cases that strictly met the inclusion criteria were included in this study, it may be possible that some dogs that had several different CNS disorders were included, such as dogs with infectious diseases and neoplastic diseases. However, the results of diagnostic imaging were highly suggestive of an inflammatory conditions in the CNS and CSF findings did not provide any evidence to suspect infectious or neoplastic CNS diseases. Furthermore, infectious CNS diseases are uncommon in our region, and most of the cases in this study showed relevant improvement with immunosuppressive therapy. The histopathological classification of GME, NME, and NLE was also impossible, and their incidences could not be predicted. Moreover, the study population was somewhat biased toward the specific breeds (e.g., Maltese, Yorkshire Terrier, and Chihuahua) that are most commonly affected by necrotizing encephalitis [15, 39, 40]. The efficacy of MMF in different histopathological types of MUE may differ, thereby affecting the results of this study, but this is beyond the scope of the present study.

Other innate limitations of this study are its retrospective nature and the lack of a control population. A placebo control group is needed to establish the accurate efficacy of combined MMF-corticosteroid treatment in MUE, but it has serious ethical problems. Therefore, a prospective, randomized, double-blinded, matched-controlled study comparing the efficacy of MMF-corticosteroid combined therapy with a corticosteroid monotherapy is considered the best option and is warranted to clarify the beneficial effects of additional MMF in MUE. The variations in therapeutic regimen among the patients due to the study’s retrospective nature might have impacted the results of this study. This includes the initial doses and timings of both MMF and prednisolone, tapering of drugs, treatment changes in relapsed patients, and doses and types of additional anticonvulsants used in some patients. Since these variances during the treatment period were inevitable, only those patients who received controlled therapy within a restricted standard were included in the study. Finally, the patients that responded well to the therapy but were still alive at the end of the study were censored from the survival data. This could probably affect the results of this study, which were analyzed with survival times. Thus, a further larger-scale study with a long-term follow-up period is warranted to substantiate these initial results.

## Conclusions

In summary, the overall findings help us extend our understanding of the MMF treatment for MUE. MMF appears to be safe and comparable to other immunosuppressive drugs for dogs with MUE. The dogs that showed a CR and did not relapse over the treatment period had significantly longer MST, suggesting that treatment should focus on the achievement of a CR and preventing a relapse for the successful management of MUE. Attention to the adverse effects including gastrointestinal upsets and sporadic infections was warranted, particularly in patients treated with high-dose, long-term MMF. However, further prospective, randomized, large-scale, controlled studies will be needed to fully understand the efficacy of MMF as an adjunctive immunosuppressive drug in dogs with MUE.

## Methods

### Case selection and data collection

The medical records of dogs diagnosed with MUE and treated with MMF and prednisolone at our hospital between May 2009 and June 2017 were retrospectively reviewed. The study included dogs with focal or multifocal CNS abnormalities, CT and/or MRI findings compatible with the focal or multifocal CNS disease most consistent with the non-infectious inflammatory change, and complete medical records containing at least one month of follow-up information after initiating MMF therapy, and meeting one of the following two criteria: (1) abnormal CSF findings consistent with inflammation without evidence of infectious or neoplastic etiology and (2) negative test results from CSF analysis for local infectious etiological agents. The dogs were excluded the following reasons: presence of concurrent systemic diseases at initial diagnosis; significant changes in treatment protocol during the study period; death associated with unknown cause during the study period.

The data collected from the medical records included information regarding signalment, body weight, the presentation of clinical signs, the interval between clinical sign onset and initial presentation, neuroanatomical lesion localization, hematology and biochemistry values, MRI and CT imaging findings, CSF analysis, treatment protocol, response, survival from the initiation of MMF therapy, and drug-related adverse events.

### Diagnostic testing

All dogs underwent a CT and/or MRI scan. MRI was performed under general anesthesia with an APERTO 0.4 Tesla scanner (Hitachi Medical Corporation, Tokyo, Japan). The standard MRI sequences for this study included T1- and T2-weighted images in the sagittal and transverse planes, fluid-attenuated inversion recovery images in the transverse plane, and T1-weighted images after the administration of intravenous gadolinium ethylenediaminetetraacetic acid injection (Omniscan; GE-Healthcare, Little Chalfont, United Kingdom) at a dosage of 0.20 mmol/kg bodyweight in the transverse planes. CT was performed under general anesthesia with a Somatom Emotion Duo scan system (Siemens medical systems, Munich, Germany). Pre-contrast and post-contrast contiguous 3-mm-thick transverse images were obtained. All post-contrast images were obtained following the injection of intravenous iohexol (Omnipaque 300; GE-Healthcare, Little Chalfont, United Kingdom) at a dosage of 0.9 g I/kg bodyweight.

Samples of CSF were collected from cisterna magna puncture during the same anesthetic session following CT or MRI scanning. The CSF samples were analyzed on the same day as sampling, and the analyses included measurement of the TNCC, measurement of TP concentration, and cytologic analysis. The results of the CSF analysis were considered abnormal if the TNCC was above 5 cells/μl and the TP was above 25 mg/dl. Cytologic CSF analysis was performed on modified Romanowsky-Giemsa-stained (Diff-Quik) cyto-centrifuged slides. Infectious disease testing for *Bartonella* spp., *Borrelia burgdorferi* sensu *lato*, *Blastomyces dermatitidis*, Canine distemper virus, *Coccidioides* spp., *Cryptococcus* spp. *Histoplasma capsulatum*, *Neospora* spp., *Toxoplasma gondii*, and West Nile virus were performed using real-time PCR assay (Neurologic RealPCR™; IDEXX Laboratories, Westbrook, ME, USA) on CSF in some of the cases.

### Treatment protocol

All patients began therapy with both MMF (CellCept®, Roche Laboratories, Nutley, NJ, USA) and prednisolone (Solondo tab, Yuhan Pharmacy, Seoul, Korea) on the day of diagnosis. Treatment was individually tailored to the basis of each patient’s clinical state; however, the following treatment protocols were strictly applied in all cases. Prednisolone was initially administered at a mean dose of 0.93 mg/kg (median, 1 mg/kg; range, 0.5–1 mg/kg) PO every 12 h. Prednisolone was continued at the initial dose for a total of four to eight weeks. After assuming a successful response, the prednisolone dose was gradually tapered to the lowest effective dose required to maintain clinical remission (reducing the dose by a quarter or half every month). The mean initial dose of MMF was 19.25 mg/kg (median, 20 mg/kg; range, 10–20 mg/kg) PO every 12 h. The MMF dose was slowly tapered to 10–15 mg/kg every 12 h after a period of CR in some cases. Drug tapering was based on the neurologic state and adverse drug effects at follow-up.

### Assessment designs

Outpatient follow-up neurological examinations and telephone communication with referring veterinarians and clients were conducted in all cases, when available, two weeks, four weeks, three months, and six months after the initiation of therapy and every six months thereafter. The neurological examinations were recorded and compared with the previous results. Complete blood counts and serum biochemistry analyses were performed at the time of initial presentation and at four to six weeks and six to twelve months following the initiation of therapy. Information regarding adverse events potentially associated with MMF treatment were collected from follow-up examinations and communication logs.

Response to therapy was defined as evidence of clinical improvement of neurologic signs at the follow-up examination and divided into three categories: (1) CR (total resolution of neurological signs); (2) PR (improvement with partially resolved neurological signs); and (3) NR (no improvement or progression of neurological signs). Relapse was defined as worsening of neurologic symptoms after an initial response had been obtained.

Response and survival-specific data for each dog were analyzed by statistical tests. All cases were divided into the “CR” and “failed CR” groups to evaluate the association of CR with survival time, and the possible risk factors for failed CR were analyzed. Furthermore, survival analysis of the dogs that showed CR within a month, the results of which would influence decision-making on treatment changes, was performed. All patients were also divided into two groups according to the interval between clinical sign onset and initial presentation, which was defined as the acuteness of the disease, and the therapeutic responses of these two groups were compared. Patients presenting with a disease course of two weeks or less after onset were defined as the “acute” group, and those presenting with a disease course of more than two weeks after onset were defined as the “chronic” group. Additionally, a paired survival assay was performed to compare dogs with a focal lesion with those showing multifocal lesions, dogs with brainstem involvement with those that did not show brainstem involvement, and dogs showing a relapse with dogs that did not.

### Statistical analysis

Pearson’s chi-squared statistic was used to test for an association between the acuteness of the disease and some response-related factors, including the occurrence of CR and relapse. Logistic regression analysis was used to test for the relationships of various risk factors to CR probability.

Kaplan-Meier survival curves for survival analysis were obtained to calculate the MST for all cases. Patients still living at the end of the study period and those who stopped the therapy during the study period and died from unrelated causes were censored from the analysis for the Kaplan-Meier curve. A log-rank statistic was used to compare various factors for survival probability. Cox’s proportional hazards regression analysis was applied to assess for the relationships of various risk factors for mortality.

Values of *P* < 0.05 were considered statistically significant. Analyses were performed with the use of SPSS software statistics (SPSS 19.0.0 for windows, IBM, Armonk, NY, USA).

## Data Availability

The datasets used and/or analyzed during the current study are available from the corresponding author on reasonable request.

## Abbreviations

CBC: Complete blood counts,
CNS: Central nervous system
CSF: Cerebrospinal fluid
CR: Complete response
CT: Computed tomography
GME: Granulomatous meningoencephalomyelitis
IMPDH: Inosine monophosphate dehydrogenase
MMF: Mycophenolate mofetil
MRI: Magnetic resonance imaging
MST: Median survival time
MUE: Meningoencephalomyelitis of unknown etiology
NLE: Necrotizing leukoencephalitis
NME: Necrotizing meningoencephalitis
NR: No response
PR: Partial response
TNCC: Total nucleated cell count
TP: Total protein
